# Toward an Improved Understanding and Treatment of Canine Mammary Tumors: Insights and Advances from the Research

**DOI:** 10.3390/ani14131890

**Published:** 2024-06-27

**Authors:** Debora Aparecida Pires de Campos Zuccari, Adriana Alonso Novais, Guilherme Henrique Tamarindo, Luiz Gustavo de Almeida Chuffa

**Affiliations:** 1 Molecular Investigation of Cancer Laboratory (MICL), Department of Molecular Biology, Faculdade de Medicina de São José do Rio Preto/FAMERP, São José do Rio Preto 15090-000, Brazil; 2 Institute of Health Science (ICS), Universidade Federal de Mato Grosso (UFMT), Sinop 78550-728, Brazil; aanovais@terra.com.br; 3 Brazilian Biosciences National Laboratory, Brazilian Center for Research in Energy and Materials (CNPEM), Campinas 13083-970, Brazil; tamarindo.gui@gmail.com; 4 Department of Structural and Functional Biology, Institute of Biosciences, UNESP—São Paulo State University, Botucatu 18618-689, Brazil; luiz-gustavo.chuffa@unesp.br

As Guest Editors of this Special Issue on canine mammary tumors, we are pleased to present a collection of articles on this highly relevant and timely topic. Mammary tumors in female dogs pose a significant challenge in veterinary medicine due to not only their prevalence but also the complexity of their pathophysiology. A deep understanding of the underlying mechanisms is crucial for developing effective diagnostic and therapeutic strategies.

Mammary tumors became prevalent in dogs two decades ago due to the improper use of contraceptives to prevent estrus and unwanted pregnancies. Initially hormone-dependent and of low malignancy, these tumors often progressed due to mammary disorganization, accumulating mutations that facilitated metastasis development and frequently resulting in the animal’s death [1,2]. Recent studies have been dedicated to unraveling the intricate molecular and cellular mechanisms driving the progression of these tumors. These research efforts have provided valuable information driving the development of more targeted and effective therapies, representing a promising advancement in addressing the challenges presented by mammary neoplasms in dogs. Among these studies, we highlight the contribution of Klopfleisch et al. (2011) [3], which offered important insights into the molecular basis of canine mammary cancer. This study, along with other recent investigations, helped shape our understanding of the pathogenesis of these tumors and offered new perspectives on therapies for and clinical management of these conditions.

The works of Goldsmith and colleagues (2011) [4] and Cassali et al. (2011) [5] also played a significant role in advancing the understanding of mammary tumors in dogs and resulted in the development of a comprehensive and accurate histopathological classification of these neoplasms, establishing an important milestone in veterinary research. By creating a standardized classification system, they provided a solid foundation for the characterization and differentiation of various types of canine mammary tumors. This contribution not only facilitated clinical diagnosis and communication among animal health professionals but also drove subsequent research on the pathophysiology, prognosis, and treatment of these diseases. The legacy left by Goldsmith and colleagues [4] continues to influence and guide current and future efforts in the fight against mammary tumors in dogs [6].

This Special Issue collates studies investigating the effect of melatonin on drug resistance in canine mammary carcinoma cells, the assessment of biosimilars as antitumor agents, the pharmacokinetics of combined treatments, and the analysis of prognostic markers. Cataldo et al. (2024) [7] demonstrate that melatonin reduced the viability of CF41.Mg spheres without additional effects when combined with cytotoxic drugs. These findings suggest a therapeutic potential of melatonin, although further research is needed to fully understand its effects on canine mammary cancer stem cells and their underlying mechanisms. Cardama et al. (2023) [8] conducted an in silico and in vitro assessment of the bevacizumab biosimilar MB02 as an antitumor agent in canine mammary carcinoma, highlighting the urgent need to develop effective therapies for these highly aggressive tumors. Machado et al. (2022) [9] investigated the pharmacokinetics of carboplatin in combination with low-dose cyclophosphamide in dogs with mammary carcinoma, while Yu et al. (2022) [10] analyzed the expression of markers such as E-cadherin, N-cadherin, vimentin, HER-2, CEA, CA15-3, and SF in the diagnosis of canine mammary tumors. Parisi et al. (2023) [11] confirmed the prognostic value of Foxp3+ cells in canine mammary tumors, while Bennett et al. (2023) [12] conducted a proof-of-concept study of an alpha-fetoprotein-derived peptide for the management of canine mammary cancer. Sgadari et al. (2023) [13] report the differential expression and subcellular localization of Sirtuin 1 between normal and malignant mammary tissue, suggesting its protective role, while Canadas-Sousa et al. (2023) found that HER2 rs24537331 also exhibits this property [14]. Rodigheri et al. (2023) report that unilateral mastectomy induces significant metabolic alterations in female dogs [15]. Additionally, important reviews addressing metabolic changes in canine mammary tumors [16] and the importance of ultrasonography in their evaluation [17] are included in this Special Issue. We recognize the growing importance of pets in our lives and society, which means that caring for their health, including the diagnosis and treatment of conditions such as mammary tumors, is vital. However, we face significant challenges when using dogs as animal models for spontaneous mammary tumor studies, especially in the American context, where most female dogs are spayed early. This limits our ability to study the disease in its natural context and may influence the results of clinical and preclinical research in countries where sterilization is not predominant. Studying canine mammary neoplasia as a model for human breast cancer is encouraged, as our animals live in the same environment as us and are exposed to the same risk factors. Therefore, it is imperative that we continue to evolve and refine our approaches in the study and treatment of mammary tumors in dogs. This includes not only advancements in understanding the pathophysiology of these neoplasms but also the comparative study of diagnostic and therapeutic methods that may be effective in non-sterilized animals. As this Special Issue is being finalized, we would like to express our gratitude to all authors, reviewers, and contributors who made this publication possible. Your efforts and contributions are instrumental to the success of this Special Issue. We hope that the articles and reviews presented here inspire new research and advancements in the understanding and treatment of canine mammary tumors. We look forward to continuing our journey of discovery and collaboration in future Special Issues. Thank you all for your support and dedication.
